# Limnological response from high-altitude wetlands to the water supply in the Andean Altiplano

**DOI:** 10.1038/s41598-021-87162-6

**Published:** 2021-04-08

**Authors:** Ignacio García-Sanz, Inger Heine-Fuster, José A. Luque, Héctor Pizarro, Rodrigo Castillo, Matías Pailahual, Manuel Prieto, Pablo Pérez-Portilla, Adriana Aránguiz-Acuña

**Affiliations:** 1 Departamento de Ciencias Geológicas, Universidad Católica del Norte, Av. Angamos 0610, Antofagasta, Chile; 2 Centro de Investigación Tecnológica del Agua en el Desierto (CEITSAZA), Universidad Católica del Norte, Av. Angamos 0610, Antofagasta, Chile; 3 Departamento de Ciencias Ecológicas, Universidad de Chile, Las Palmeras 3425, Santiago, Chile; 4 Departamento de Geología, Universidad de Chile, Casilla 13518 Correo 21, Santiago, Chile; 5 Departamento de Química, Universidad Católica del Norte, Av. Angamos 0610, Antofagasta, Chile; 6 Departamento de Ciencias Históricas y Geográficas, Universidad de Tarapacá, Av. 18 de Septiembre 2222, Arica, Chile; 7 Departamento de Biología, Facultad de Ciencias, Universidad de Tarapacá, Av. 18 de Septiembre 2222, Arica, Chile

**Keywords:** Biodiversity, Wetlands ecology, Environmental sciences, Limnology

## Abstract

The Andean Altiplano-Puna is located at an elevation of approximately 4000 m.a.s.l. and is delineated by the Western and the Eastern Andes Cordillera. The high-altitude wetlands (HAWs) in the Central Andes are unique ecosystems located in the Altiplano that provide many ecosystem services. The objective of this study was to characterize the spatial heterogeneity of the environmental conditions associated with varying hydrology of the HAW, Salar de Tara, in the Andean Altiplano. Sediment samples of up to 20 cm in depth were obtained from various salt flat sub-environments. The samples were analyzed using proxies for mineralogical and chemical composition, thermal analysis, and magnetic susceptibility. Diatom and ostracod communities were also identified and analyzed. The results reflected changes in the geochemistry, carbon content, mineralogy, and magnetic properties of the sediments that can be explained by variations in the sources of water input to the Salar de Tara. The sub-environments depend on the supply of water via the groundwater recharge of springs adjacent to the streamflow from the Zapaleri River, which promotes greater diversity and richness of genera. Our results suggest that water extraction at industrial levels greatly impacts the persistence of hydrologically connected HAWs, which concentrate a worldwide interest in brine mining.

## Introduction

The Andean Altiplano-Puna is a highland surface located at an elevation of approximately 4000 m.a.s.l., between 15° and 28°S, and is delineated on either side by the Western and Eastern Andes Cordillera^1^. In contrast to the extreme aridity of the Atacama Desert lowlands, in the Altiplano area, wetter conditions with concentrated precipitation occur during the austral summer (December to March) due to the South American monsoon^2^. During the rest of the year, precipitation is uncommon. The seasonal moisture source of the Altiplano-Puna plateau is explained by the displacement of the Inter-Tropical Convergence Zone (ITCZ) to the south^3^ and by a low-pressure continental zone formed over the Gran Chaco region in Argentina during the summer months. This phenomenon forces easterly winds in the Amazon to be redirected to the south, transporting significant humidity to the sub-tropics^4^. The arrival of moist winds contributes to the rainfall recharge of lakes, aquifers, and high-altitude azonal wetlands and salt flats^5^. Additionally, in the Altiplano zone, strong interannual variability is associated with the El Niño Southern Oscillation (ENSO) phenomenon, resulting in fluctuations between warm and dry phases in El Niño years, and cold and wet phases in La Niña years^3,6^.

The high-altitude wetlands (HAWs) in the Central Andes are unique ecosystems that correspond to areas of land that are fully or partially covered with shallow water permanently or during certain periods of the year. These wetlands include peatland (locally known as *bofedales*), wet meadows (locally known as *vegas*), and salt flats. The HAWs are hotspots of biodiversity, have a high degree of endemism^7,8^, control the water cycle^9^, and are an integral component of the global climate system^10,11^. Additionally, they have important cultural value for local agropastoralist indigenous communities^12^. Although the HAWs play an essential role in the lives of the inhabitants and ecological sustainability, numerous climatic and anthropogenic factors threaten these ecosystems. First, the salt flats are located in an area that is extremely vulnerable to climate change^13^. Observations and projected scenarios for the Andes suggest alterations in precipitation patterns and evaporation rates that will affect the restricted water availability for these ecosystems^14,15^. Second, the extractive industries located in the region use surface water and groundwater for mining activities, development, and urban consumption. Historically, copper mining has resulted in the over-extraction of water from the highlands^16^ and, since the early 1980s, lithium mining has resulted in the over-extraction of brines which has depleted the aquifers and threatened local biodiversity^17^. The area of HAW is located within the “Lithium Triangle,” a region of the Andes (including parts of Argentina, Bolivia, and Chile) that contains greater than half of the world’s lithium reserves. This situation is exacerbated by a water management system based on private rights and markets^18^ and the current lithium boom.

Due to various temporal scales that alternate between aridity and humidity, the Altiplano-Puna water bodies, lakes, salt flats, wet meadows, and peatlands located in the highlands of South America are informative archives of the evolution of the socio-environmental and climatic conditions in the region and their impacts on the biota^19,20^. On a millennial scale, wetter to drier periods have been identified from lake sediments using a multiproxy approach that included the analysis of isotopic and magnetic properties and the study of biological proxies such as fossil plant cover and diatom assemblages, as recorded from the Salar de Uyuni sediments^21,22^ and Lake Titicaca^23,24^. For shorter timescales, decadal phenomena such as the ENSO have been identified through sedimentary records^25^, as have recent anthropic disturbances including the intensive mining activities in the Atacama Desert on lacustrine sediments and the biotic communities of the Inca Coya Lake^26,27^ and the Ascotán salt flat^28^.

**Figure 1:**
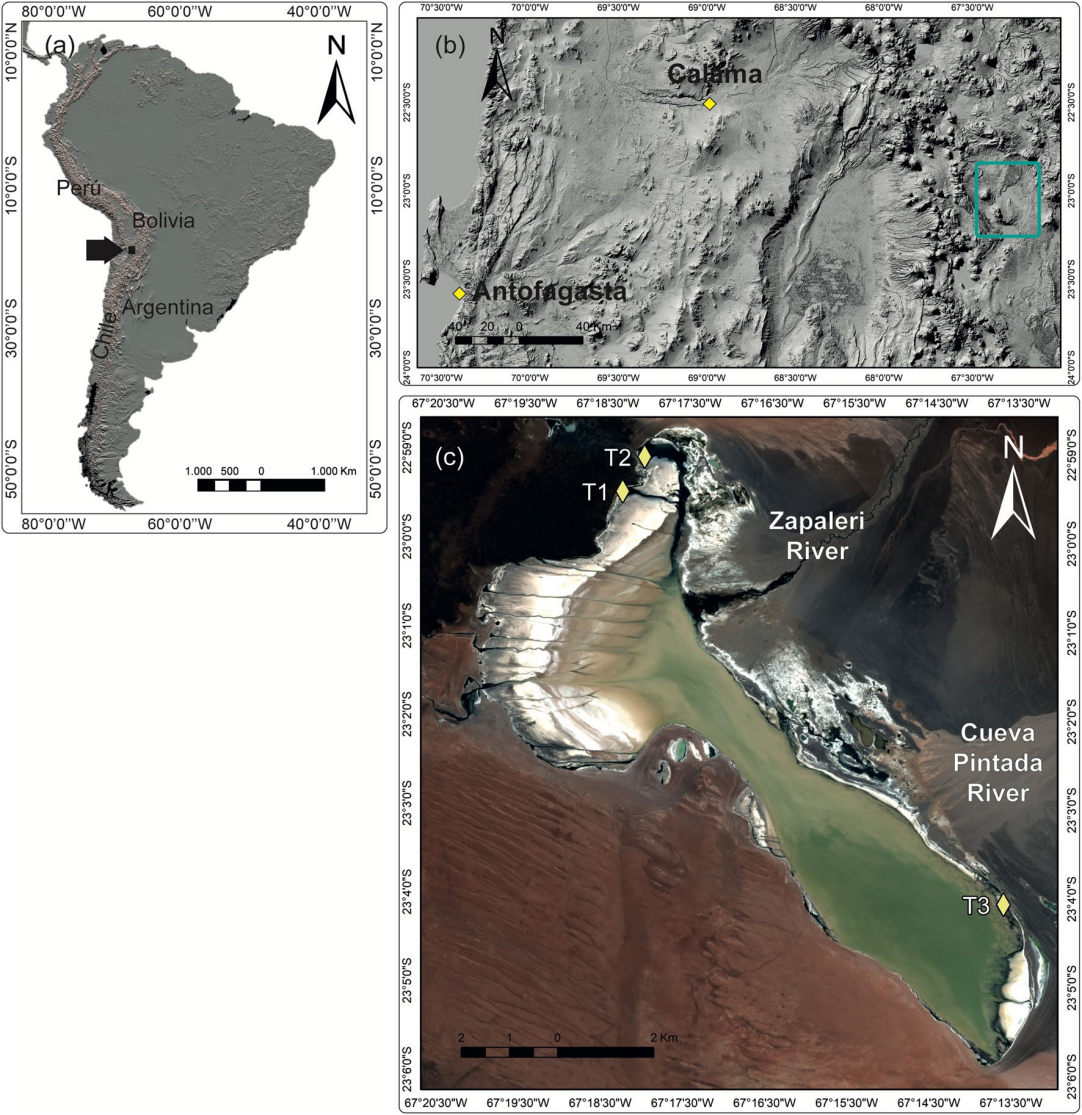
Salar de Tara. Location of Salar de Tara in Andean Altiplano-Puna, and sampling points distributed in the basin (**a**) ASTER Global Digital Elevation Model (GDEM) V.3 from 2006 was retrieved on 2020/06/16 from https://lpdaac.usgs.gov, maintained by the NASA EOSDIS Land Processes Distributed Active Archive Center (LP DAAC) at the USGS Earth Resources Observation and Science (EROS) Center . https://doi.org/10.5067/ASTER/AST14DEM.003; (**b**) Sentinel-2A, European Space Agency (ESA) image courtesy of the U.S. Geological Survey. https://doi.org/10.5066/F76W992G; (**c**) Shuttle Radar Topography Mission Products, Jet Propulsion Laboratory, NASA (Date: 2000–02-11T00:00:00.000Z), http://www.jpl.nasa.gov/srtm/).

The objective of this study was to characterize the spatial heterogeneity of the environmental conditions associated with the varying hydrology of the HAW, Salar de Tara, in the Andean Altiplano. Water and sediment records were analyzed using several approaches, such as geochemical, thermal, and mineralogical analyses. Moreover, magnetic properties were utilized to identify the content and dynamics of the sediment transport and biological proxies (communities of diatoms and ostracods), and to examine the impact of the spatial heterogeneity on several ecological descriptors. We studied the Salar de Tara as a model (Fig. 1), which is located in a territory claimed by the Atacameño (Likan antai) indigenous community of Toconao, a protected area of Chile and a Wetland of International Importance (as determined by the Ramsar Convention). Despite this conservation status, the Chilean State has granted mining rights for the exploitation of lithium and other mineral resources, thus, threatening the future of these ecosystems and the indigenous communities that claim their ancestral rights.

Our results highlight the inconsistency of the physical, chemical, and biological environments of HAWs in regions with variable hydrology, which may be associated with the fluctuating humidity in the Altiplano system. Furthermore, the results of this study emphasize the relevance of these factors in the formation of aquatic communities.

## Results

### Water parameters and sediment sampling

Three sampling points were selected to capture the heterogeneity of the landscape: an upwelling spring (T1), a salty lagoon (T2), and a playa lake (T3) (Fig. 1). The results of the physicochemical analysis of water are presented in Table 1. The alkalinity was similar at all three sampling points, and higher pH levels were observed at T3. The electric conductivity was lower at T1, which was closest to an upwelling zone. The water temperature was approximately 8 °C at T1 and T2, which was attributed to the discharge of shallow groundwater. The higher temperature observed at T3 (18 °C) was likely associated with the atmospheric temperature because this sampling point consisted of a shallow playa lake (< 15 cm depth, approximately).

**Table 1:** Water parameters obtained in situ from sampling points in Salar de Tara.

Sampling Points	Conductivity (µS/cm)	pH	Temperature (°C)
T1	1768	8.40	10.4
T2	3455	8.13	17.1
T3	3747	8.93	18.7

### Mineralogical and elemental composition of the sediments

**Figure 2:**
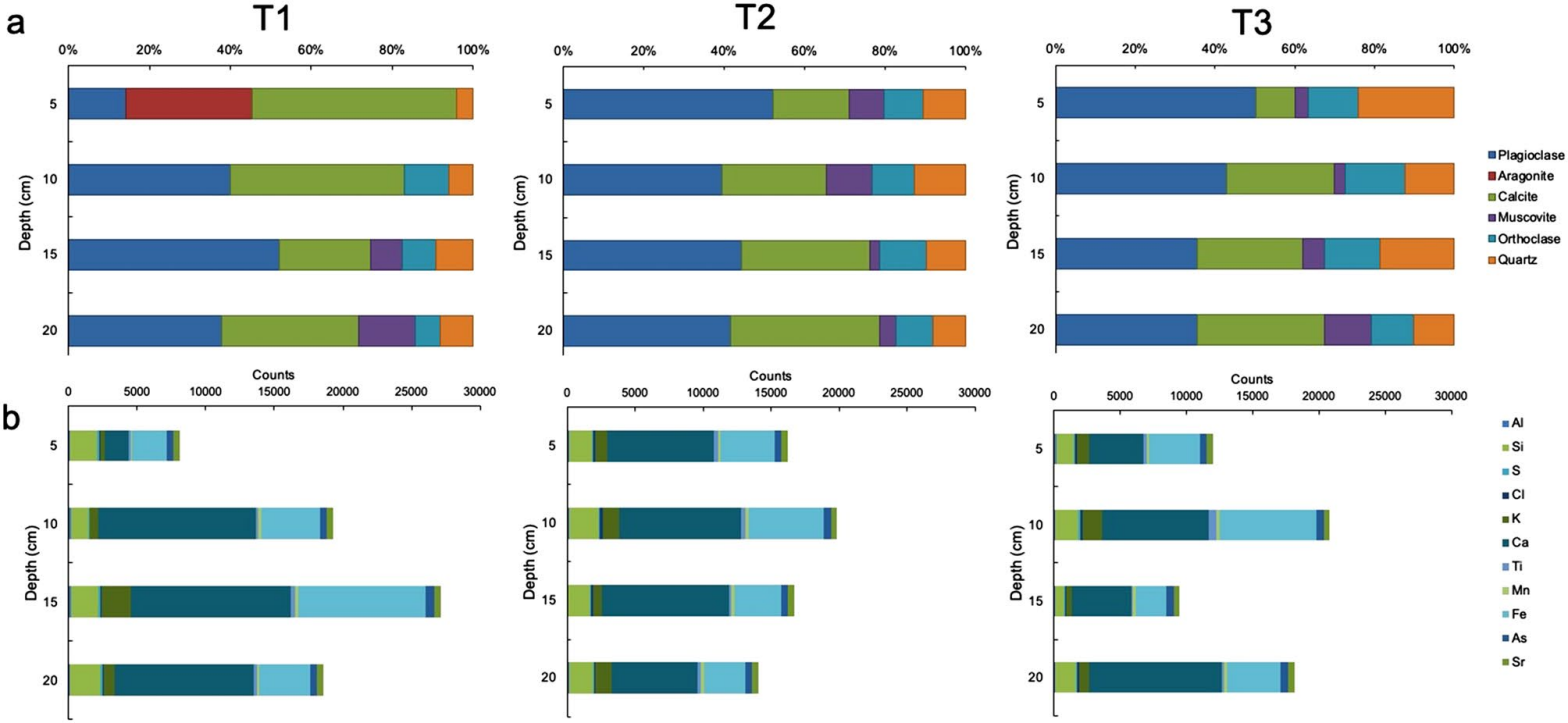
Mineralogy and Geochemistry of sediments. (**a**) Percentual composition of minerals and (**b**) Elemental composition (measured in counts) identified in sampling points in Salar de Tara T1, T2 and T3, through 20 cm depth records.

The mineral phases identified from the sampling points showed differences in the current sediment composition between the sites, and changes were observed in the percentage of minerals from the oldest to the most recent deposits (Fig. 2a). For T1, the samples obtained from the upper layer had the most dissimilar composition among the samples, with a greater proportion of calcite (50.56%) and aragonite (31.16%) and lower proportions of plagioclases and quartz. The sediment obtained from a depth of 5 to 10 cm was dominated by carbonates of calcite and tectosilicates of albite, with a minor presence of orthoclase. The samples obtained between depths of 10 and 20 cm showed a similar composition, with a dominance of plagioclases-type andesine and Mg-rich calcite, which together comprised greater than 70% of the composition. The samples obtained from the T2 sampling point showed a more homogeneous composition through the depth layers, with a greater proportion of plagioclases and calcite (which jointly represented ~ 65–80%) and a lower proportion of muscovite, orthoclase, and quartz. In the T2C sample, obtained between a depth of 10 and 15 cm, andesine was replaced by albite (44.26%). For T3, the dominance of plagioclases decreased while the percentage of calcite increased with soil depth. Lower and variable concentrations of quartz and orthoclase, and increasing concentrations of muscovite were measured and, combined, did not exceed 40% throughout the record.

In summary, the samples revealed a predominance of carbonates and tectosilicates, except for the upper layer of sediment (0–10 cm) at the T1 sampling point, and all the samples contained a minimal amount of phyllosilicates. The samples collected from T1 were dominated by aragonite and calcite (> 80%), primarily in the upper sediments. This finding is possibly associated with recent authigenic processes. Conversely, the samples collected from T2 and T3 showed a greater proportion of tectosilicates at all depth layers, which is likely associated with detrital accumulation.

The elemental composition was consistent with the mineralogical profile (Fig. 2b and Supplementary Table). For T1, the allochthonous proportion increased from the upper to the bottom layer, indicating increased Al, Si, and Ca. This trend was associated with the trend of increasing plagioclases with depth. The presence of orthoclase provided K. Additionally, Fe was likely detected from ferric oxides, indicating a high correlation with the occurrence of plagioclases. Therefore, the detrital origin of the sediment triggered an increase in Al, Si, Ca, K, and Fe. In contrast, the authigenic minerals (i.e., calcite and aragonite) were associated with the upper layer of the profile, and they resulted in low values in the detected counts during the analysis. Moreover, relatively high values were associated with tectosilicates.

For T2 and T3, the same geochemical elements were also detected (Al, Si, Ca, K, and Fe), although in more homogeneous proportions. Samples obtained from T2 showed the most homogeneous profile, and no significant changes were observed with depth. This result indicates that allochthonous minerals (plagioclase, orthoclase, and quartz) are predominant in the sediment. Samples collected from T3 revealed part of an authigenic layer at 0 cm and 15 cm depth due to decreased values of Al, Si, Ca, K, and Fe. Overall, the elemental composition was associated with the geochemical signature of the catchment area, as the watershed is comprised of quartz, plagioclase, orthoclase, and ferric oxides. Several trace elements were also detected (e.g., S, Mn, and As). Hence, the analysis did not reveal the internal facies of the Salar de Tara (i.e., sulfate and chloride) since the concentrations of S and Cl were extremely low or absent. Finally, As was detected in all the samples collected and was homogeneously distributed through the depth profiles, suggesting that this geochemical element constitutes a natural background provided by the volcanic origin of the Chilean Altiplano.

**Figure 3:**
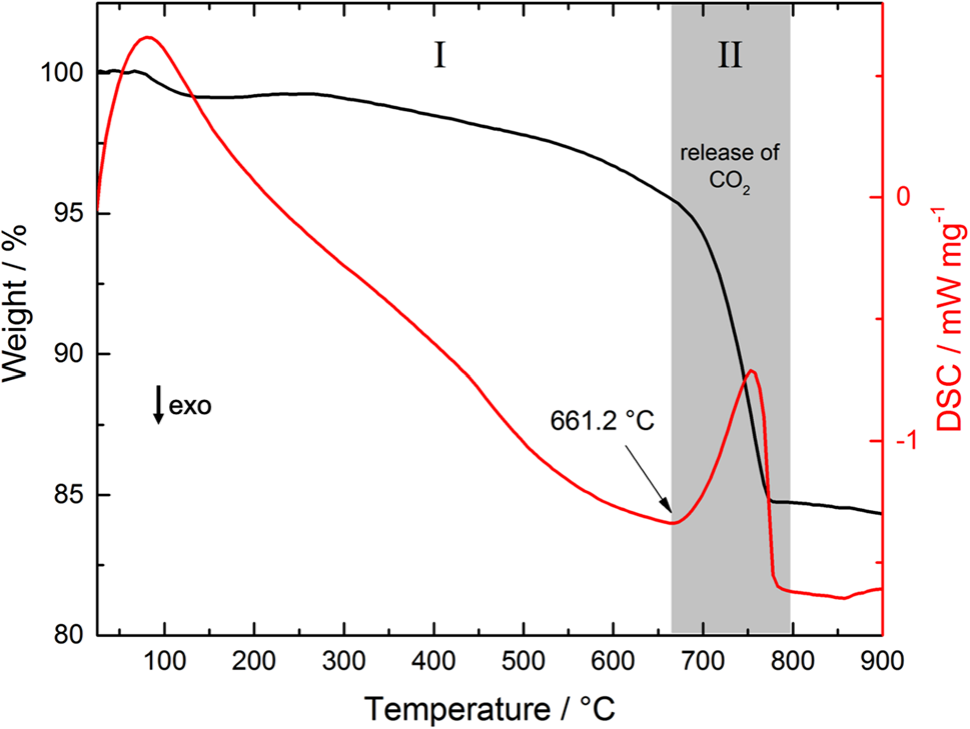
TG-DSC. TG-DSC curves of sample T1C, indicating the onset temperature of the endothermic event corresponding to the release of CO_2_. Similar thermograms are observed for the remaining samples.

The thermogravimetry and differential scanning calorimetry (TG-DSC) technique was used to obtain an estimator of carbon content in the sediment samples (Fig. 3). The obtained thermogram was divided into two regions. In the first region, ranging from room temperature to 661.2 °C, the samples experienced a constant mass loss of approximately 6%, which was attributed to the intrinsic humidity of the samples and the dehydration of silicate hydrate minerals. In the second region, a substantial change in the slope of the TG curve began at 661.2 °C (onset temperature). This change was accompanied by an endothermic effect in the DSC curve, which indicated the decomposition of the carbonates in the sample into CO_2_ and the respective oxides. A similar result was observed for the remaining samples. Assuming that calcium carbonate was the sole source of carbonate, the amount was estimated to be 35.48%. The results obtained for all samples are summarized in Table 2. The samples obtained from the T1 sampling site demonstrated the highest carbonate content in the upper layer sediments, which also corresponded to the highest carbonate content among all the samples analyzed. The samples collected at T2 and T3 revealed increasing carbonate content through the depth profiles, with more homogeneous values for T2.

**Table 2:** Results obtained from TG-DSC analysis. Temperature onset is the temperature when the decomposition of carbonates starts, and Mass loss correspond to the amount of released CO_2_.

Sampling point	Depth (cm)	Temperature onset (°C)	Mass loss (%)	CaCO_3_ (%)
T1	5	653.9	22.90	52.09
	10	683.9	13.28	30.21
	15	661.2	15.60	35.48
	20	663.7	15.08	34.30
T2	5	662.4	12.83	29.18
	10	691.1	14.84	33.76
	15	681.0	15.08	34.30
	20	680.2	18.29	41.60
T3	5	664.8	9.48	21.56
	10	691.4	15.06	34.26
	15	701.7	21.37	48.61
	20	689.5	19.93	45.33

### Analysis of sediment magnetic properties

**Figure 4:**
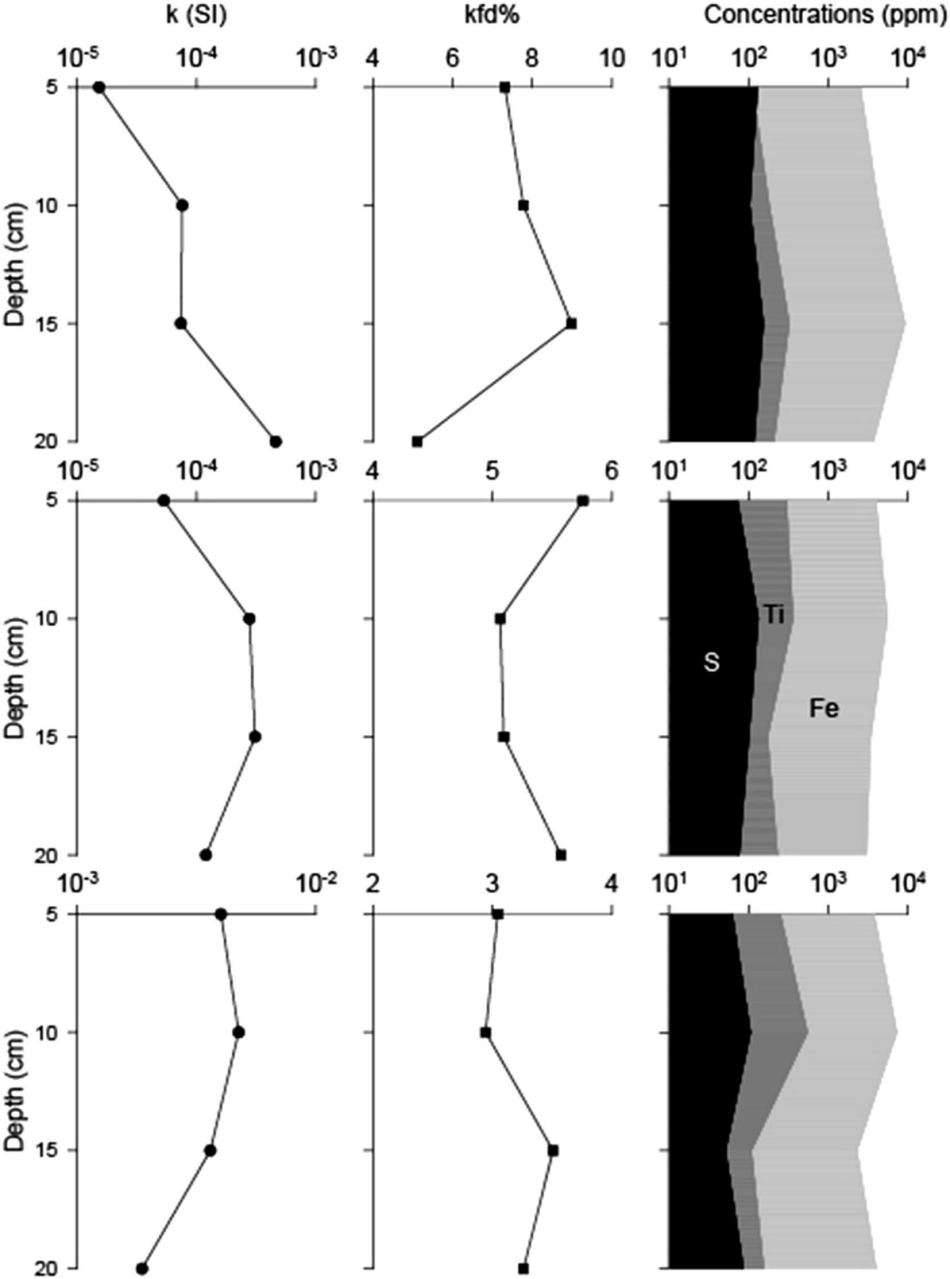
Magnetics parameters. Bulk magnetic susceptibility values measured at low frequency (*k*), Frequency-dependent magnetic susceptibility (*k*fd%) values and S, Ti and Fe concentrations (ppm) versus the depth of the different study sites. From top to bottom: sampling site T1, T2 and T3.

Figure 4 illustrates the variations of bulk magnetic susceptibility (*k*) and frequency-dependent magnetic susceptibility (*k*fd %) with respect to the Fe, Ti, and S content in the stratigraphic records of the various study sites. The *k* values varied from 4.76 × 10^–3^ (SI) in the sediment layers of T3 to 1.53 × 10^–5^ (SI) in the sediment layers of T1, with an average value of ca. 1.30 × 10^–3^ (SI). The highest *k* values were measured in the samples collected from T3, with an average value of 3.56 × 10^–3^ (SI) whereas the lowest *k* values were detected in the samples obtained from T1 and T2 (with average values of 1.57 × 10^–4^ and 1.91 × 10^–4^, respectively) (Fig. 4). Moreover, at these sites, *k* values of two orders of magnitude lower were detected in several samples. For all sites, the *k* values tended to be higher in the middle portion of the depth profile except for T1 which demonstrated a high *k* value at the bottom.

The *k*fd % parameter was used to qualitatively estimate the presence and concentration of small ferromagnetic particles with magnetic sizes near the superparamagnetic/single-domain (SP/SD) limit^29,30^. The *k*fd % values varied from 9.0% in the sediment layers at T1 to 2.9% in the sediment layers at T3, with an average value of 5.3%. In this case, the highest *k*fd % values were observed in samples obtained from T1 and T2 (with average values of 7.3% and 5.4%, respectively) whereas the lowest *k*fd % values were detected in samples collected from T3, with an average value of 3.2%. Based on these results, we propose that the number of extremely small ferromagnetic particles was only significant in layers from sites T1 and T2, which may reflect the presence of authigenic processes over magnetic minerals^30–32^.

The *k* values were primarily influenced by the concentration of ferromagnetic minerals. The Fe and Ti curves were comparatively similar for all sites and these minerals were present in relatively high concentrations, suggesting that the primary magnetic phase in the sediments may be (titano)magnetite and/or (titano)maghemite, which coincides with the ferromagnetic minerals recorded in surrounding volcanic rocks^28,32^. No significant variations were observed in the composition throughout the sections. Additionally, for T3, the observed similarities between the Fe, Ti, and S curves may indicate the presence of iron sulfides in this sector. For T1, the Fe and Ti curves were similar to the *k*fd % curves, which indicates that in this sector, the magnetic signal is primarily influenced by variations in the magnetic particles of authigenic origin.

Thus, the magnetic signal detected at T1 was influenced by a high concentration of ferromagnetic minerals of authigenic origin, while the magnetic signal observed at T3 was mainly influenced by ferromagnetic minerals of detrital origin. A mixture of both types of particles (but with a greater predominance of particles of authigenic origin) influenced the magnetic signal at T2. Based on the results, it was also inferred that the highest concentration of ferromagnetic minerals occurred in the middle portions of each stratigraphic section.

### Biological proxies and statistical analyses

A single order of the class Ostracoda was identified, namely the Podocopida, 97% of which was represented by members of the Ciprididae family. The dominant genus was *Cyprinotus* (90%) and the genus *Herpetocypris* represented only 7%. The Lymnocytheridae family was represented by a single genus, *Limnocythere* (3%). The three genera that were identified showed a similar distribution in the T1 and T2 cores but not in the T3 core, wherein the abundance of individuals was too low to be representative of the ostracods in the area.

**Figure 5:**
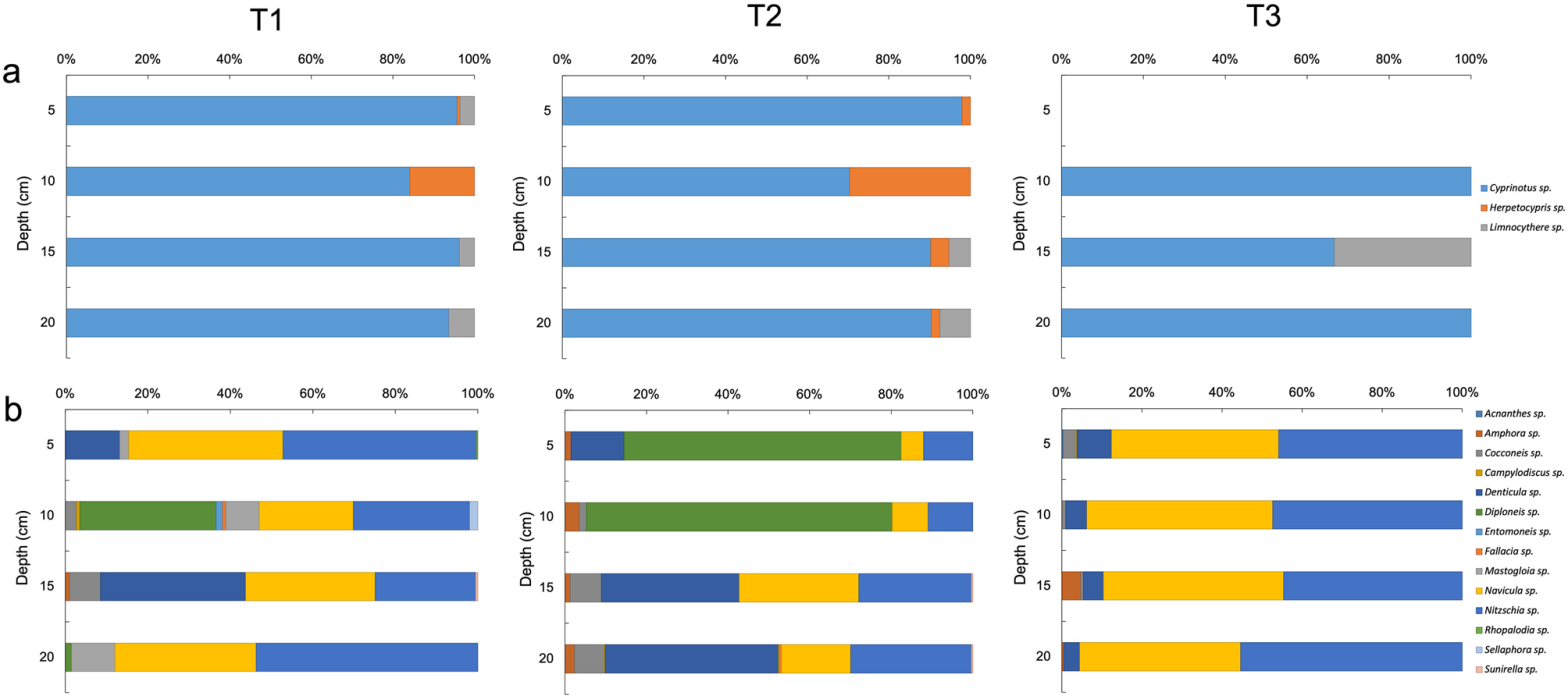
Relative abundance of diatoms and ostracods. (**a**) Percentual abundance of ostracods genera and (**b**) diatoms genera identified in sampling points in Salar de Tara T1, T2 and T3, through 20 cm depth records.

Only benthic taxa of the class Bacillariophyceae (diatoms) were recorded. The distribution and representation of the genera identified at the three sampling points are presented in Table 3 and Fig. 5. The genera richness and diversity index calculated based on the relative abundance of the diatom and ostracod genera varied within and between the sites and demonstrated similar patterns. Sampling point T1 showed greater richness and diversity in the 5–10 cm layer. Below that layer, the richness and diversity decreased with depth, was the poorest and least diverse in the bottom layer (15–20 cm). Conversely, T2 showed a regular pattern of increasing richness and diversity with depth. The lowest and most homogeneous values of genus richness were observed throughout all depths at T3. A similar result was obtained for diversity, except for the surface layer, in which the diversity values were slightly higher than that observed at T2.

**Table 3:** Abundance of Ostracoda and diatoms identified, and community indices calculated from Salar de Tara samples.

	Genus	T1				T2				T3			
		A	B	C	D	A	B	C	D	A	B	C	D
**Ostracoda**	Cyprinotus sp.	945	931	910	714	875	567	847	510	0	16	4	14
	Herpetocypris sp.	7	175	0	0	18	238	42	12	0	0	0	0
	Limnocythere sp.	35	0	35	48	0	0	49	42	0	0	2	0
**Diatoms**	Acnanthes sp.	0	0	0	0	0	0	0	0	2	0	0	0
	Amphora sp.	0	0	6	0	7	16	8	14	0	0	28	2
	Cocconeis sp.	0	14	43	0	0	8	47	42	16	4	4	0
	Campylodiscus sp.	0	4	0	0	0	0	0	2	2	0	0	0
	Denticula sp.	72	0	202	0	62	0	208	250	46	22	30	16
	Diploneis sp.	0	171	0	8	322	342	0	2	0	0	0	0
	Entomoneis sp.	0	8	0	0	0	0	0	0	0	0	0	0
	Fallacia sp.	0	4	0	0	0	0	0	4	0	0	0	0
	Mastogloia sp.	12	42	0	56	0	0	0	0	0	0	0	0
	Navicula sp.	204	118	180	182	26	40	180	100	222	193	268	162
	Nitzschia sp.	256	146	140	286	57	50	170	175	244	197	266	223
	Rhopalodia sp.	2	0	0	0	0	0	0	0	0	0	0	0
	Sellaphora sp.	0	10	0	0	0	0	0	0	0	0	0	0
	Sunirella sp.	0	0	3	0	0	0	2	2	0	0	0	0
	Simpson 1-D	0.571	0.634	0.599	0.624	0.530	0.686	0.657	0.725	0.607	0.588	0.602	0.561
	Shannon H	1.167	1.426	1.27	1.227	1.058	1.353	1.44	1.585	1.081	1.035	1.099	0.967

**Figure 6:**
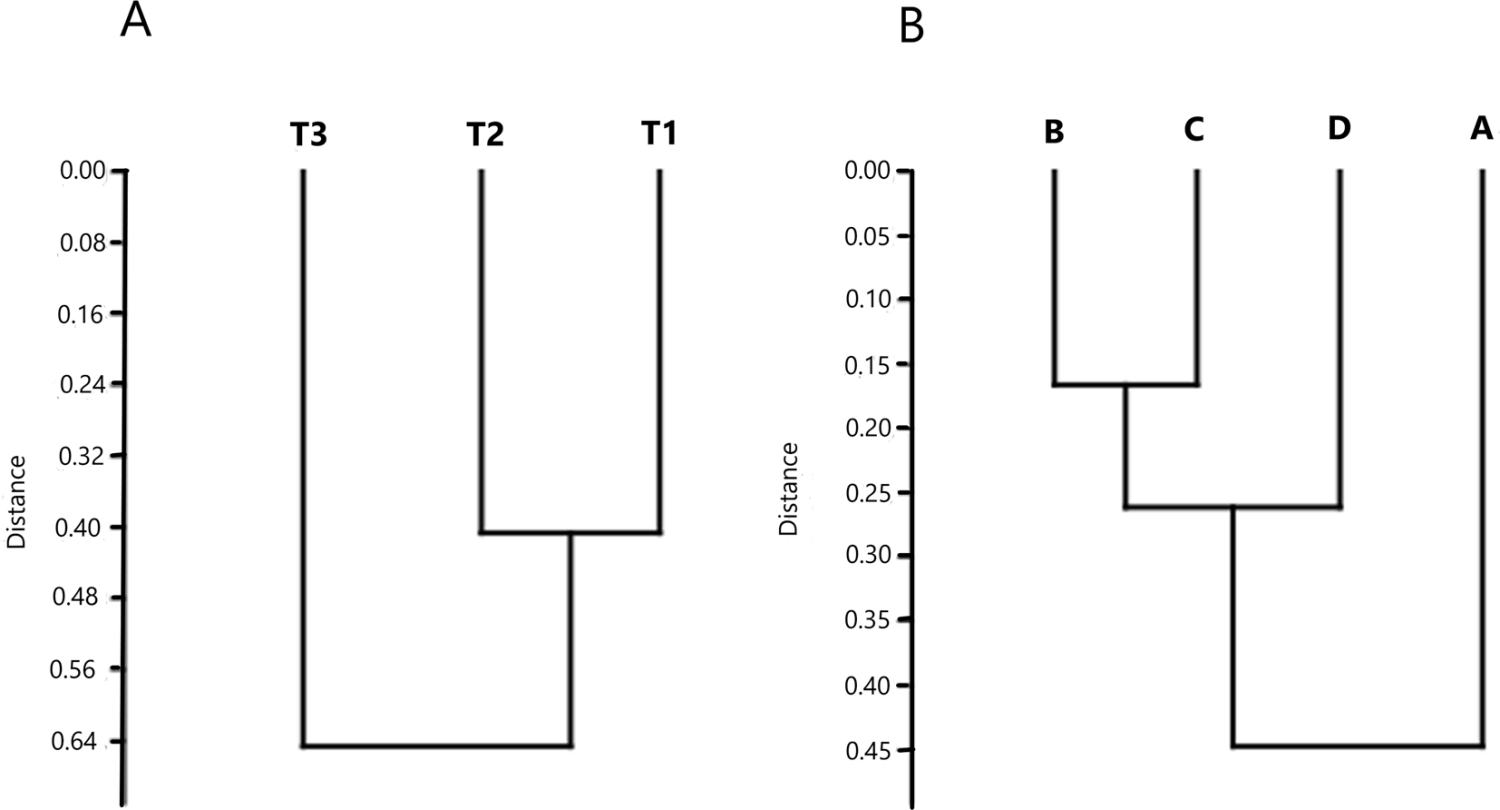
Cluster of community structure. Cluster analysis showing the similitude in Euclidean distance between sampling points from Salar de Tara and between depth layers.

Two clusters were obtained by comparison using the Shannon–Wiener diversity index, for each stratigraphic level (mode R) from a sampling point (mode Q) (Fig. 6). T1 and T2 showed similar levels of diversity, with mean values of 1.27 and 1.36, respectively. These two sites were separated from T3 which had a lower mean diversity value (1.04). This ordination mode had a correlation coefficient of 0.896. The clusters obtained from stratification levels (mode R) grouped more tightly in layers B (5–10 cm) and C (10–15 cm), with an average diversity value of 1.27. The greatest distance was identified between this group and the deepest level, D (15–20 cm). The upper level (A layer) showed the most differentiation compared with the rest of the layers and revealed the lowest mean diversity. The correlation coefficient in this mode was 0.73.

The results of a multivariate analysis of variance with permutations (PERMANOVA) showed no significant interaction between space and depth, however, the community indices differed significantly among the sampling points (F = 5.33; d.f. = 1; *p* = 0.017).

## Discussion

The sediment proxies of past change recorded in the cores analyzed during this study demonstrated variability over time concerning the input of water to the Salar de Tara in the Altiplano-Puna plateau of the Andes Cordillera. These results are likely associated with changes in precipitation and hydrology.

Spatial and temporal heterogeneity was observed between and within the samples. Carbonates (aragonite-calcite) and tectosilicates (plagioclases) were dominant at the T1 sampling site, primarily in the upper layer of sediments (> 80%). Moreover, a greater proportion of carbonates was observed in the upper-layer sediment samples and may be associated with an authigenic origin. This idea is reinforced by the observed high *k*fd% values, suggesting the presence of small magnetic Fe-oxides of authigenic origin^31,32^. At the T3 sampling site, carbonate concentrations showed a decreasing trend and an inverse relationship with tectosilicates, which may be associated with increased detrital input. The observed agreement between the highest *k* and lowest *k*fd % values at T3 indicated a high Fe-Ti oxide content of detrital origin^32^. Finally, intermediate carbonate and tectosilicate concentrations were observed at the T2 sampling site, which supported the observed intermediate *k*fd percentage (> 3%) and low *k* values, demonstrating lower concentrations of carbonates and Fe-oxides of authigenic origin than at T1. Thus, the sediments obtained from T1 were primarily composed of minerals of authigenic origin, while at T3, the sediments are mostly composed of allochthonous minerals. The sediments collected from T2 contained a mixture of both types of particles, however, the particles of detrital origin were dominant.

The Salar de Tara is the only salt flat in the region where permanent surface water flows converge. The main source of surface water for the Salar de Tara is the inflow from the Zapaleri River and the secondary Cueva Pintada River (Fig. 1)^33^. Therefore, during wet periods, the rivers supply low-salinity water^33^ and sediment to the shoreline of the salt flat, triggering the subsequent development of alluvial fans. Two alluvial fans are located along the eastern shoreline of the salt flat and are associated with both rivers. The presence of allochthonous minerals in samples from T2 and T3 may be associated with climatic fluctuations due to the hydrological behavior of the Zapaleri and the Cueva Pintada Rivers, respectively. The results obtained for T3 strongly support this theory, as the mineral content of detrital origin in the samples collected from this site was greater, and the concentration of authigenic minerals was less than in the samples obtained from T1 and T2. This is in accord with the larger alluvial fan generated by the Cueva Pintada river.

Arsenic was detected in all the samples collected and showed a homogeneous distribution throughout the profiles, suggesting that this geochemical element constitutes a natural background provided by the volcanic origin of the Chilean Altiplano, as has been stated for other HAWs of the Altiplano-Puna^34^.

Variations in mineralogical analysis, thermal analysis, and an analysis of the magnetic properties of the sediments were consistent with the observed distribution of biota. The composition of ostracod and diatom genera showed variations at numerous levels for the T1 and T2 sampling points which may be explained by environmental factors such as the water period and seasonality^35–37^. An ostracod of the genus *Cyprinotus* was dominant and has been associated with regular changes in the water table, such as cyclical floods of minor magnitude. Nevertheless, an increase in diversity at the subsurface level coincided with the presence of ostracods of the genus *Herpetocypris* which are most associated with more stable water levels over time^38,39^. Ostracods of the genus *Limnocythere* were scarce. This observation may be due to the fact that members of this genus are more successful in deeper ponds^40^. These sedimentary layers would be associated with periods under the influence of the rainfall of the austral summer. In the subsurface layer (5–10 cm depth), samples from T1 showed the highest genera richness and biodiversity. Conversely, samples from T3, a characteristic shallow playa lake, revealed the lowest richness and diversity.

Diatom analysis showed that *Achnanthes*, a genus of low-profile diatoms, was exclusively observed in the upper layer of sediment at T3. This may be due to the fact that this site was located in an evaporitic area with a playa lake, a very shallow water column, and absent vegetation. Previous studies on Altiplano diatoms have demonstrated associations between periphytic diatoms and low water levels, while tychoplanktonic, facultative planktonic, and planktonic diatoms have been associated with higher water levels^41,42^*.* A lack of dating controls prevents us from drawing conclusions about the observed similarities in temporal variations among the cores, however, we can conclude that the variability in the ostracod and diatom genera was greater among the sites than within each site. This reflects the heterogeneous landscape of the study region and suggests that multiple cores from a variety of locations may be required to accurately assess long-term environmental change.

The salt flat exhibited sub-environments where the water supply is primarily dependent on the climate, which controls the groundwater flow and the streamflow. The salinity of the various areas of the HAWs is influenced by direct precipitation, runoff, riverine input, and groundwater^43^. The outlier condition of the T1 sampling point is most likely associated with the impact of the water supply from complementary sources (springs related to groundwater recharge and the streamflow from the Zapaleri River), which supply the characteristic water and sediment that influence the high diversity and genera richness observed at this site. Our results highlight the relevance of the water inflows, organic matter, and salts for the maintenance and structure of the ecological communities and ecosystems in the HAWs.

The Andean Altiplano-Puna constitutes a recharge zone for the regional aquifer in the study area^44,45^. From this highland surface, located at an elevation of approximately 4000 m.a.s.l., the groundwater flows towards the west to the Salar de Atacama, at approximately 2500 m.a.s.l. When the groundwater from the Altiplano-Puna reaches the Salar de Atacama, a mixing zone is generated between the lithium-rich brine and the fresh water. This triggers the formation of lacustrine systems along the eastern border of the Salar de Atacama^46–48^. Paleoenvironmental studies have reported fluctuations in the input of water recharge in the Salar de Atacama during the Pleistocene^49^. Hence, the identification of this hydrogeological variability is also crucial for understanding the human settlement along the Salar de Atacama. Our results concerning the limnological characteristics of the Salar de Tara may be associated with environmental processes related to the recharge of water in the Andean Altiplano-Puna. Thus, paleolimnological records may help to infer how the fluctuations of groundwater flux influence the brine and the formation of other lacustrine systems of ecological interest.

Nevertheless, industrial interests regarding brine mining pose a risk for the Salar de Tara. Several lithium mining projects are expected to be developed in Tara and the surrounding salt flats (e.g., Quisquiro, Pujsa, Aguas Calientes, and Helada), and the lithium-rich brine of the Salar de Atacama is the primary objective of the mining activity^46,50,51^. This situation threatens the stability and conservation of the hydrogeological characteristics and ecological communities in the basin, and it also impacts the indigenous communities that claim territorial rights in the area and that have already been affected by colonialism and industrial mining^52^.

In the Los Flamencos National Reserve, which includes the Salar de Tara, only certain key areas are protected under a weak institutional model, including lakes, salt flats, and the Tamarugo forest. Sonter et al.^53^ stated recently that this is a consideration of worldwide conservation interest. While several protected areas prevent mineral activities, greater than 14% of these areas contain mines within or near their boundaries, and the consequences for biodiversity may extend a great distance from the core mining sites. Furthermore, the generation of renewable energies may exacerbate other non-climatic changes that impact the conservation of biodiversity. For example, the Salar de Tara and all the salt flats included in the lithium triangle are exposed to brine and freshwater extraction at industrial levels. Conservation efforts focused on landscapes, biodiversity, and indigenous practices may be applied with greater success if the authorities include integral regional considerations of hydrogeological and ecological connectivity between the protected patches that constitute fragmented land(water)scapes^54^ and local ecological knowledge from the indigenous communities and their territorial demands^55^.

## Methods

### Study area

The Salar de Tara (22° 01′ 59″ S, 67° 16′ 30″ W, 4,300 m.a.s.l.) is located in the highlands of the Atacama Desert, known as the Altiplano-Puna plateau (Antofagasta Region, northern Chile). The total extent of this basin is 1,710 km^2^ and it is hydrogeologically shared by Bolivia, Argentina, and Chile, with an area of 1,209 km^2^, and a depositional area of 32 km^2^^56^. The topography of the sector is of an endorheic basin, with rock outcrops and a regular cone shape. The volcanic system is comprised of lava flows, tuff, and basaltic to andesitic breccia of the Pliocene–Pleistocene age^57^. Within the study area, groundwater flows through the volcanic system from northwest to southeast and is finally intercepted by the topographical profile. The drainage network that runs through the east of the basin includes the Zapaleri River and the Cueva Pintada River, while the west drains into the Chico and Huailitas Rivers. Therefore, the Salar de Tara receives a relevant contribution of surface water from the Zapaleri River which has an exceptional flow in the arid system of the Andes in northern Chile.

The Salar de Tara contains the largest number of species of birds and mammals observed in the wetlands of the Antofagasta Region, which makes it a focus of great biological conservation interest^58^. Among the species of avifauna, there are three species of high Andean flamingos, and the endemic species *Liolaemus molinai* represents the reptiles. The biodiversity of mammals, reptiles, and birds is supported by microbiota, diatoms, and benthic microinvertebrates in the lacustrine sediments^59^.

Climatic features affect the density and diversity of vegetation, and plant cover decreases as the altitude and latitude increase^58^. The salt flat is located in an altitudinal vegetation belt dominated by perennial herbs and several cushion plants^58^. Additionally, the azonal plant communities develop what is locally known as *vega salina*, the Andean high-altitude wetland type, composed mainly of *Baccharis acaulis*, *Distichlis humilis*, *Deyeuxia velutina var*. *atacamensis*, *D. crispa, Mulinu, crasifolium, Oxychloe, Carex marítima*, *Triglochin concinna*, *Puccinellia frigida*, *Stipa frigida, Fabiana bryoides, Parastrephia quadrangularis*, and *Adesmia sp*.^57,59^. Many of these species provide year-round fodder to domesticated and wild South American camels (llamas [*Lama glama*], and vicuñas [*Vicugna vicugna*]).

Human occupation of the area began in the Early Archean period (ca. 9000 to 6000 BC)^60^. Currently, local Atacameño communities use the area as grazing fields and consider it a sacred landscape. This is why the Atacameño community of Toconao claims the area. Furthermore, due to its socioecological relevance, the Salar de Tara is part of the National System of Wild Protected Areas (more specifically, part of the Los Flamencos National Reserve) and is protected under the RAMSAR convention on wetlands.

The fieldwork was conducted in April 2018, after the seasonal summer rains. Three sampling points were selected: an upwelling spring (T1), a salty lagoon (T2), and a playa lake (T3) (Fig. 1). These sites were selected, based on a preliminary analysis of aerial images, to include the heterogeneity of the waterscapes of the highland salt flats and in response to the interests expressed by the Atacameño community of Toconao.

### Water parameters and sediment sampling

At each sampling point, the physicochemical parameters of the surface water (temperature, conductivity, and pH) were measured in situ with a multi-parameter meter (Hanna HI98194).

The sediment samples were obtained using a Lenz bottom sampler with a grasping area of 225 cm^2^, which allowed the total sample volume to be separated into 5 cm layers using dividing sheets. Samples from four layers were obtained at each sampling point and were labeled as A (0–5 cm depth), B (5–10 cm depth), C (10–15 cm depth), and D (15–20 cm depth), resulting in a total of twelve sediment samples. The samples were deposited in plastic bags, sealed, and refrigerated at 4 °C until laboratory analysis.

### Mineralogical and elemental composition of the sediments

The mineralogical and elemental analyses of the sediment samples were conducted in the Unidad de Equipamiento Científico MAINI at the Universidad Católica del Norte, Chile (UCN), and the thermal analysis was conducted in the Chemistry Department of UCN.

The first step in identifying the mineral phases of the samples was to pulverize sediment sub-samples in an agate mortar to eliminate the roughness of the surface of the samples. Next, the samples were sieved on a 53 µm sieve to homogenize the grain size. X-ray diffraction (XRD) analysis was conducted using a Bruker D8 Advance with a Cu X-ray tube (λ = 1.5406 Å) which was operated at 40 kV. The phase identification and quantification were performed using EVA (Bruker AXS. EVA, version 10; Bruker AXS, Karlsruhe, Germany, 2004) and TOPAS (Bruker AXS. TOPAS, version 3; Bruker AXS, Karlsruhe, Germany, 2005) software, respectively.

X-ray micro fluorescence analysis was performed to determine the elemental composition of the samples. Dry samples with a grain size of 53 microns were used and were mounted on double-sided carbon fiber tape which was then attached to a slide. The aligned samples were then placed on the platform of the TORNADO Micro-XRF M4 Spectrometer (Bruker nano GmbH) for analysis.

Thermal analysis of the sediments was performed using a simultaneous TG-DSC apparatus (Netzsch STA 448 Jupiter F3). The sediments were roughly powdered, and approximately 15 mg of each of the homogenous samples was used in the analysis. The samples were loaded into alumina crucibles and heated from room temperature to 900 °C at a heating rate of 10 °C min^−1^, under an N_2_ atmosphere with a flow rate of 50 mL min^−1^. Finally, the samples were cooled to room temperature. Onset temperatures of energetics events were evaluated by taking the first derivative of the DSC curve.

### Analysis of sediment magnetic properties

A sediment fraction (ca. 2.00 g) was mounted into 8 cm^3^ standard size paleomagnetic cubes for volume magnetic susceptibility (*k*) measurements. The *k* was measured in SI units using a Kappabridge MFK1-FA susceptibility bridge (AGICO Co.) under normal ambient conditions (22–24 °C) and a 200 A/m field. Measurements were performed at (a) 976 Hz (*k*f1) and (b) 15.616 Hz (*k*f3). This method allowed us to calculate the frequency-dependent magnetic susceptibility (*k*fd %)^30^, as follows:

1 $$kfd\% = (\left( {kf1 - kf3} \right)/kf1) \times 100$$

in which *k*fd % is widely used as a normalized parameter, and it is interpreted to reflect the content of the particles close to the SP/SD boundary.

### Biological proxies and statistical analyses

To remove the large material (such as branches and stones) from the sample, 500 mL of each sediment sample was filtered through a 5 mm mesh. Next, the samples were filtered through a 500 µm and 250 µm diameter mesh to separate invertebrate organisms of the class Ostracoda. To identify the genera of the separated ostracods, 200 mL of clean sample was observed under a binocular stereomicroscope, following the protocol described in the relevant literature^61–64^. A minimum of 300 shells of individual ostracods per sample was obtained to ensure the validity of the statistical analyses and an accurate representation of the fauna present^65^. Diatoms (Bacillariophyceae) were observed in pre-treated sediments by digestion of the organic matter and carbonates with 30% H_2_O_2_ and 37% HCl^66^. Dilutions were conducted to observe an estimated 400 organisms per sample. The samples were prepared for taxonomic analysis as indicated in Díaz et al. (2016) and identified to genus level by observation under a microscope (Leica DM750) and Camera Mod ICC 50 HD, following ad-hoc literature^66,67^.

Community indices, including the genera richness (S), exponential of Shannon entropy (Exp(H’)), Pielou (J), and Shannon and Simpson Hill’s ratio index (HE and DE, respectively) were calculated by site and depth. The sampling sites were classified by HCR cluster analysis (in Euclidean distance) to observe the similitude in both Q mode (between samples) and R mode (between layers from samples), following the unweighted pair-group average model (UPGMA). Cluster analyses were obtained using the free software PAST^68^.

A principal component analysis (PCA) was conducted to assess the relative importance of each measured environmental variable on the community indices of species richness, exponential of Shannon entropy (Exp(H’)), Pielou, Shannon, and Simpson Hill’s ratio index by site and depth. The PCA was conducted in the R environment using the *vegan* package^69^.

To determine whether there was a spatial and temporal association in the variation of the biotic community, a multivariate analysis of variance with permutations (PERMANOVA) was performed^70^. This analysis was applied to the triangular Bray–Curtis similarity matrix and was considered an experimental design based on the existence of different levels of one or more factors. This type of analysis performs a partition of the variance components in relation to the factor(s) to be considered and calculates a pseudo-F-statistic^70^ considering 9,999 permutations. In the present study, we considered two factors: space (three sites) and time (four depth levels). The PERMANOVA was conducted in the R environment using the *vegan* package^69^.

## Supplementary Information

Supplementary Information
