# Author Correction: Limnological response from high-altitude wetlands to the water supply in the Andean Altiplano

**DOI:** 10.1038/s41598-021-96407-3

**Published:** 2021-08-16

**Authors:** Ignacio García‑Sanz, Inger Heine‑Fuster, José A. Luque, Héctor Pizarro, Rodrigo Castillo, Matías Pailahual, Manuel Prieto, Pablo Pérez‑Portilla, Adriana Aránguiz‑Acuña

**Affiliations:** 1 Departamento de Ciencias Geológicas, Universidad Católica del Norte, Av. Angamos 0610, Antofagasta, Chile; 2 Centro de Investigación Tecnológica del Agua en el Desierto (CEITSAZA), Universidad Católica del Norte, Av. Angamos 0610, Antofagasta, Chile; 3 Departamento de Ciencias Ecológicas, Universidad de Chile, Las Palmeras 3425, Santiago, Chile; 4 Departamento de Geología, Universidad de Chile, Casilla 13518 Correo 21, Santiago, Chile; 5 Departamento de Química, Universidad Católica del Norte, Av. Angamos 0610, Antofagasta, Chile; 6 Departamento de Ciencias Históricas y Geográfcas, Universidad de Tarapacá, Av. 18 de Septiembre 2222, Arica, Chile; 7 Departamento de Biología, Facultad de Ciencias, Universidad de Tarapacá, Av. 18 de Septiembre 2222, Arica, Chile

Correction to: *Scientific Reports* 10.1038/s41598-021-87162-6, published online 08 April 2021

In the original version of this Article, Adriana Aránguiz-Acuña was incorrectly affiliated with ‘ANID Millennium Science Initiative Program Nucleo Milenio UPWELL, Av. 18 de Septiembre 2222, Casilla 6-D, 1000000, Arica, Chile’. The correct affiliation is listed below.

Departamento de Biología, Facultad de Ciencias, Universidad de Tarapacá, Av. 18 de Septiembre 2222, Arica, Chile

The original Article has been corrected.

