# Supplementary material for: Limnological response from high-altitude wetlands to the water supply in the Andean Altiplano
Source: Sci Rep. 2021 Apr 8;11:7681. doi: 10.1038/s41598-021-87162-6 (PMC8032802; doi:10.1038/s41598-021-87162-6)
Supplement: Supplementary file 1 — Supplementary Information [file 41598_2021_87162_MOESM1_ESM.docx]

Limnological response from high-altitude wetlands to water supply in the Andean Altiplano

Ignacio García-Sanz, Inger Heine-Fuster, José A. Luque, Héctor Pizarro, Rodrigo Castillo, Matías Pailahual, Manuel Prieto, Pablo Pérez-Portilla, Adriana Aránguiz-Acuña

**Supplementary Table S1**. Elemental composition of sediment samples obtained from different points in Salar de Tara by µXRF analysis.

| Sampling point | Depth (cm) | Element (counts per second) | | | | | | | | | | |
| --- | --- | --- | --- | --- | --- | --- | --- | --- | --- | --- | --- | --- |
|  |  | Al | Si | S | Cl | K | Ca | Ti | Mn | Fe | As | Sr |
| T1 | 5 | 59 | 2030 | 129 | 110 | 320 | 1762 | 112 | 112 | 2550 | 439 | 445 |
|  | 10 | 153 | 1260 | 103 | 40 | 578 | 11487 | 181 | 208 | 4298 | 460 | 505 |
|  | 15 | 182 | 2012 | 153 | 69 | 2129 | 11642 | 323 | 261 | 9279 | 605 | 509 |
|  | 20 | 135 | 2215 | 117 | 60 | 790 | 10171 | 208 | 209 | 3696 | 514 | 438 |
| T2 | 5 | 160 | 1678 | 72 | 196 | 854 | 7812 | 299 | 234 | 3985 | 488 | 428 |
|  | 10 | 148 | 2148 | 132 | 184 | 1224 | 8921 | 360 | 230 | 5543 | 520 | 435 |
|  | 15 | 109 | 1565 | 100 | 149 | 657 | 9330 | 173 | 196 | 3440 | 527 | 495 |
|  | 20 | 138 | 1740 | 76 | 112 | 1181 | 6381 | 231 | 208 | 3042 | 502 | 485 |
| T3 | 5 | 157 | 1345 | 62 | 189 | 906 | 4089 | 249 | 195 | 3792 | 514 | 506 |
|  | 10 | 142 | 1717 | 105 | 186 | 1462 | 8065 | 546 | 306 | 7310 | 528 | 459 |
|  | 15 | 62 | 732 | 51 | 78 | 434 | 4491 | 108 | 221 | 2308 | 560 | 453 |
|  | 20 | 106 | 1554 | 86 | 125 | 802 | 9970 | 155 | 298 | 4021 | 576 | 462 |
